# Maternal Accommodation of Adolescent Body Dysmorphic Disorder: Clinical Correlates and Association with Treatment Outcomes

**DOI:** 10.1007/s10578-024-01754-7

**Published:** 2024-09-03

**Authors:** E. Hogg, G. Krebs, D. Mataix-Cols, A. Jassi

**Affiliations:** 1 Research Department of Clinical, Educational and Health Psychology, University College London, 1-19 Torrington Place, London, UK; 2 Institute of Psychiatry, Psychology & Neuroscience, King’s College London, De Crespigny Park, Denmark Hill, London, UK; 3 National and Specialist OCD, BDD and Related Disorders Clinic for Young People, South London and Maudsley NHS Foundation Trust, London, UK; 4 Centre for Psychiatry Research, Department of Clinical Neuroscience, Karolinska Institutet & Stockholm Health Care Services, Region Stockholm, Stockholm, Sweden; 5 Department of Clinical Sciences, Lund University, Lund, Sweden

**Keywords:** Body dysmorphic disorder, Family accommodation, Maternal accommodation, Cognitive behavioural therapy

## Abstract

Family accommodation (FA) is widely-recognised as an important clinical phenomenon in obsessive–compulsive disorder (OCD) and anxiety disorders, and is related to poorer treatment outcomes. However, FA has not been quantitatively explored in Body Dysmorphic Disorder (BDD). The aim of this study was to investigate the patterns and correlates of maternal accommodation in adolescent BDD, and its association with treatment outcomes. Participants were 131 adolescents with BDD and their mothers who completed the Family Accommodation Scale—Parent Report (FAS-PR) as part of routine clinical practice in a National and Specialist Service for Young People at the Maudsley Hospital, London. Seventy-six (58%) young people received specialist cognitive behavioural therapy for BDD and had post-treatment data available. All mothers engaged in at least one form of accommodation. Providing reassurance (98.5%) and assisting avoidance (88.5%) were the most commonly endorsed behaviours. Levels of accommodation were positively associated with clinician-rated BDD symptom severity (*r* = 0.18*, p* = 0.041) and maternal symptoms of anxiety, depression and stress (*r* = 0.41*, p* < 0.001), and negatively associated with child global functioning (*r* = –0.38*, p* < 0.001). Maternal accommodation did not predict treatment outcomes (β = 0.055, *p* > 0.05). The findings indicate that maternal accommodation is common and has important clinical correlates, but does not impact on treatment response. Consequently, the relationship between maternal accommodation and BDD symptoms may differ to that evidenced in paediatric OCD. Future longitudinal research exploring maternal and paternal accommodation, and assessing variables of interest at multiple time-points throughout treatment, is needed to advance understanding of the role of FA in adolescent BDD.

**Supplementary Information:**

The online version contains supplementary material available at 10.1007/s10578-024-01754-7.

## Introduction

Body Dysmorphic Disorder (BDD) is characterised by a preoccupation with perceived flaw(s) in one’s physical appearance that appear non-existent or slight to others, and associated avoidance and repetitive behaviours aimed at checking, camouflaging, or correcting the perceived flaw(s) [1, 2]. BDD usually emerges during adolescence [3], with prevalence estimates of 1.7–3.6% in this age group [4–6]. BDD is associated with poor social, educational and family functioning, comorbid psychopathology, and concerningly high rates of suicidality [7–12].

Cognitive behavioural therapy (CBT) and serotonin reuptake inhibitors (SSRI) are the recommended treatments for BDD [13]. There is emerging evidence for CBT in the treatment of adolescent BDD, with response rates of between 40% in a pilot randomised controlled trial (RCT; [10]) to 79% in a naturalistic study of multimodal treatment consisting of CBT with or without pharmacotherapy [14]. While these outcomes are promising, they highlight that a substantial proportion of adolescents with BDD do not respond to existing treatment approaches. Identifying factors associated with treatment outcomes has the potential to inform clinical decision-making but may also shed light on mechanisms that maintain BDD, which could in turn inform the development of modified protocols to enhance treatment outcomes. Current evidence on predictors of CBT outcomes in BDD are highly inconsistent (e.g. [14–19]). This is likely due to a number of factors, including the fact that previous studies have often had modest sample sizes and therefore limited statistical power [18]. However, another possibility is that certain key factors that may be associated with treatment response have been overlooked to date. One such factor, which has been extensively examined in a closely related disorder, obsessive–compulsive disorder (OCD), is family accommodation (FA).

FA refers to behavioural changes made by family members (often parents/carers) with the intention of reducing the OCD sufferer’s distress and/or supporting their present functioning. Examples of accommodation include providing reassurance, participation in rituals, assisting avoidance of feared objects or situations, and following desired routines [20]. FA in paediatric OCD is commonplace, with up to 99% of parents reporting they accommodate their child’s OCD symptoms (e.g. [21–23]). FA is clinically important because higher levels of accommodation are associated with greater OCD symptom severity (e.g. [21, 22, 24]), increased functional impairment (e.g. [22, 25, 26]), greater depressive symptoms [22], and greater symptoms of parental psychological distress (e.g. [22, 26, 27]).

Furthermore, higher levels of FA have been found to predict poorer treatment outcomes for young people with OCD (e.g. [22, 28]). From a cognitive-behavioural perspective, FA is formulated as a compulsion which provides a temporary relief of anxiety but inadvertently maintains anxiety and OCD symptoms in the long-term. Consequently, FA is hypothesised to impair learning opportunities and obstruct the effectiveness of exposure and response prevention (E/RP) components in treatment [29, 30].

Only one study to date has explored FA in adolescent BDD. Jassi et al. [31] interviewed five adolescents with BDD, their parents, and clinicians working in a specialist BDD clinic to explore if FA was relevant to the clinical presentation. Qualitative data indicated that all participants reported FA of BDD symptoms, and the behaviours described were comparable to those observed in OCD, such as reassurance giving/seeking, participation in rituals and facilitating avoidance. However, this study was small, and did not quantitatively explore the relationship between FA, clinical variables and treatment response. Quantitatively investigating FA in a large sample is important to support our understanding of whether FA is significantly associated with the clinical presentation of BDD and treatment outcomes, and should therefore be addressed and targeted in treatment.

In summary, there is substantial evidence to indicate that higher levels of FA are associated with a more severe clinical presentation and poorer treatment outcomes in OCD, but FA has been largely overlooked in relation to BDD in youth. The aim of the current study was to provide the first quantitative examination of FA and its clinical correlates in BDD. The first aim was to explore the frequency and patterns of maternal accommodation, with the hypothesis that there would be high rates of maternal accommodation of BDD. The second aim was to examine demographic and clinical correlates of maternal accommodation and it was hypothesised that a) child age and sex would not be associated with maternal accommodation, b) BDD symptom severity, depressive symptoms, and maternal distress would show positive associations with maternal accommodation, and c) global functioning would show a negative association with maternal accommodation. The third and final aim was to examine associations between maternal accommodation at assessment and CBT outcomes. It was hypothesised that higher levels of maternal accommodation at assessment would predict poorer treatment outcomes (i.e. smaller reductions in BDD symptom severity).

## Method

### Participants

This study utilised routinely collected data from a clinical cohort of patients from the National and Specialist OCD, BDD and Related Disorders Clinic for Young People at the Maudsley Hospital, London, and data from a RCT conducted in the same clinic [10]. The sample consisted of 131 young people (aged 13 to 18) who met the Diagnostic and Statistical Manual of Mental Disorders, Fifth edition (DSM-5, [1]) criteria for BDD, and their mothers. For parent-report measures, we focused specifically on maternal report since: a) mothers completed measures more frequently than fathers; and b) previous research in OCD has shown that mothers tend to show higher levels of accommodation than fathers [22].

All 131 young people were offered treatment and 76 (58%) of these received CBT for BDD and had post-treatment data available. The remainder did not have post-treatment data available because they were waiting for treatment or in treatment at the time of data analysis (n = 10, 8%), or they did not engage in any treatment sessions and/or did not complete post-treatment measures (n = 45, 34%). Of these 45 young people, 12 did not accept treatment (27%), 26 accepted treatment but did not engage (58%), and 7 completed treatment but did not have post-treatment data available (15%). There were no significant differences in age, BDD severity (clinician and self-reported), global functioning, depressive symptoms, maternal accommodation, and maternal distress between participants who had clinician-administered post-treatment data available versus those who did not (all *p* > 0.05; see Table S1 of supplement). However, there was a significant difference in sex, such that females were more likely than males to have post-treatment data available (*p* = 0.043). Approval for the study was obtained from the Health Research Authority, Health and Care Research Wales Authority (Reference: 22/HRA/1731) and the South London and Maudsley Child and Adolescent Mental Health Service Research Committee. As described above, a sub-sample of young people took part in a RCT, which was approved by the National Research Ethics Service Committee South East Coast—Kent (REC reference 11/LO/1605).

### Measures

*Body Dysmorphic Disorder – Yale-Brown Obsessive Compulsive Scale for Adolescents* (BDD-YBOCS-A; [32]). The BDD-YBOCS-A is a 12-item semi structured clinician administered interview to assess the severity of BDD symptoms during the past week. The total score ranges from zero to 48, with higher scores indicating greater symptom severity. The measure has good psychometric properties, including being sensitive to changes in BDD severity [22, 32]. Good internal consistency was evident in the current sample (Cronbach’s α = 0.85).

*Children’s Global Assessment Scale* (CGAS; [33]). The CGAS is a single item measure completed by the clinician to assess severity of disturbance to functioning. The score ranges from 1 to 100, with higher scores representing healthier levels of overall functioning. The CGAS has good psychometric properties [33, 34].

*Appearance Anxiety Inventory* (AAI; [35]). The AAI is a 10-item self-report measure of cognitive and behavioural processes associated with BDD during the past week. The total score ranges from zero to 40, with higher scores indicating greater appearance-related anxiety. The measure has sound psychometric properties [35, 36]. Mastro et al. [37] suggested a cut-off score of 20 for clinically significant BDD symptoms. In the present study, Cronbach’s α was 0.81.

*Mood and Feelings Questionnaire Child-Version* (MFQ-C; [38]). The MFQ-C is a 33-item self-report measure of depressive symptoms in young people between 6 and 19 years of age. The total score ranges from zero to 66, with higher scores on the measure indicating greater depressive symptoms, and scores of 27 or higher potentially indicating the presence of depression. The measure has been demonstrated to have good psychometric properties [38, 39]. Cronbach’s α was 0.95 in the current study.

*Family Accommodation Scale – Parent Report* (FAS-PR; [40]. The FAS-PR is a modified parent-report version of the semi-structured, clinician administered FAS [41]. The original measure includes 13-items, however, item five was removed for the purpose of this study as this question refers to OCD. All other items are phrased in a way that is suitable for BDD (e.g. referring to compulsive behaviours) and were therefore retained. Items are completed by parents on a Likert scale of 0–4. The FAS-PR assesses family members’ behavioural involvement in their child’s BDD and the degree of family distress and interference associated with such involvement. A higher score on the measure is reflective of higher levels of accommodation.

Since the FAS-PR has not previously been used with BDD samples, an exploratory factor analysis (EFA) was undertaken to identify the factor structure of the measure. Promax rotation was used in the EFA to allow potential factors to be correlated. Both the visual scree-plot (see Figure S1 in the supplementary information) and the eigenvalue > 1 criteria indicated a two-factor solution, retaining all 12 items, which accounted for 59.6% of the variance. Retained items and factor loadings for each factor are provided in Table S2 in the supplementary information. Factor one was labelled ‘modified routines’ (FAS-PR-MR) and included five items which described maternal adaptations to their own and the families’ routines, as well as the subsequent impact on their own distress. Factor two was labelled ‘involvement in rituals’ (FAS-PR-IR) and included seven items which described maternal reassurance, their direct role in the child’s BDD-related rituals, and the child’s response when mothers were not involved in rituals. Cronbach’s α for the FAS-PR total score was 0.91, indicating excellent internal consistency. Internal reliability of the two subscales (modified routines and involvement in rituals) was good (Cronbach’s alphas 0.89 and 0.83, respectively).

*Depression Anxiety Stress Scale* (DASS; [42]). The DASS is a validated 42-item self-report measure that assesses the core symptoms of depression, anxiety, and stress over the last week. The total score ranges from zero to 168, with higher scores indicating a greater severity of psychological distress. The DASS was completed by mothers as a measure of maternal psychological distress and the Cronbach’s α was 0.98.

### Procedure

Young people and their parents attended an initial diagnostic assessment with a specialist multidisciplinary BDD team. During the three-hour assessment, the BDD-YBOCS-A was completed with young people, and separately, parents reported a detailed account of current difficulties and developmental history. BDD-YBOCS-A interviews were conducted by clinical psychologists with specialist knowledge in BDD, or assistant and trainee clinical psychologists who received training in the measure and close supervision. The multidisciplinary team discussed all BDD-YBOCS-A interviews and parental accounts, and a DSM-5 [1] diagnosis of BDD was assigned based upon the information obtained. Young people and their parents completed all self-report measures ahead of the initial assessment and at the end of treatment. Clinicians conducted BDD-YBOCS-A interviews at the end of treatment.

### Treatment

Seventy-six (58%) of the young people had post-treatment data available after receiving CBT for BDD at the clinic (mean number of CBT sessions = 17.49; SD = 5.74; range = 5–34). The CBT intervention was protocolised [10, 14] and delivered by trained clinical psychologists or trainee clinical psychologists under close supervision from senior psychologists with specialist expertise. Young people attended weekly sessions, and treatment included psychoeducation on BDD and anxiety, the development of a hierarchy of compulsions and avoidance behaviours, E/RP and relapse prevention. Whilst mothers were encouraged to join their child in at least the initial psychoeducation sessions, involvement of mothers throughout treatment, and the extent to which maternal accommodation of BDD symptoms was explicitly addressed, varied based upon the individual case formulation.

Over half of the 70 young people who received CBT were also taking SSRI medication during treatment (n = 40; 53%). Given there was often a wait between the diagnostic assessment and treatment commencing, most young people were on a stable medication dose when they started CBT. There were no significant differences between participants who were taking SSRI medication during treatment and those who were not with respect to any of the variables investigated in the study (all *p* > 0.05; see Table S3 of supplement).

### Statistical Analyses

Descriptive statistics were used to examine levels and patterns of maternal accommodation at initial assessment. Clinical and demographic correlates of maternal accommodation were examined initially using Pearson correlations, and variables found to be significantly associated with FAS-PR scores were entered into multivariable linear regression models to investigate independent and significant associations with the FAS-PR total and subscale scores.

A linear regression model was performed to test the association between maternal accommodation of BDD symptoms at assessment and CBT treatment outcomes. FAS-PR total score at assessment was entered as the predictor (independent variable), and the outcome (dependent variable) was post-treatment BDD-YBOCS-A total score. The model controlled for baseline BDD severity (BDD-YBOCS-A), child age, child sex and any other variables found to be uniquely associated with FAS-PR scores in the analyses of the assessment data. G*Power [43] calculations indicated the number of participants necessary to achieve power of 0.80 to detect significant associations between assessment maternal accommodation and post-treatment BDD symptom severity, with four variables entered as predictors and a Cohen’s *f*^2^ effect size of 0.25, was 52. All analyses were conducted using SPSS Version 29. Missing data was handled using individual mean substitution where sufficient item-level data was completed for variables (at least 80% of items; [44]). Where sufficient item-level data was not completed, cases were excluded from analyses pairwise.

## Results

Demographic and clinical characteristics of the sample are provided in Table 1. The majority of young people were female (n = 108; 82%) and White British (n = 75; 74%). The mean age was 15.65 years (SD = 1.40) and the retrospectively reported mean age of the onset of BDD symptoms was 12.30 years (SD = 2.39). The mean BDD-YBOCS-A score was 34.73 (SD = 5.46) and the mean FAS-PR total score was 24.50 (SD = 11.78). The mean CGAS score was 41.00 (SD = 8.76), suggestive of a moderate degree of impairment in functioning in most social areas or severe interference with functioning in one area.

**Table 1:** Demographic and clinical characteristics of the sample (N = 131)

Variable (n)	n	%
Sex (131)		
Female	108	82.44%
Male	23	17.56%
Ethnicity (101)		
White British	75	74.26%
Other white unspecified	6	5.94%
Black British	5	4.95%
White and black Caribbean	3	2.97%
Any other group	3	2.97%
White and black African	2	1.98%
White and Asian	2	1.98%
Turkish	1	0.99%
Black and white	1	0.99%
Chinese and white	1	0.99%
British Asian	1	0.99%
Other latin American	1	0.99%
SSRI at assessment (131)		
Yes	63	48.09%
No	68	51.91%
School attendance (102)		
Yes	41	40.20%
No	25	24.51%
Part-time	23	22.55%
Home educated	13	12.75%
ASD diagnosis (65)		
Yes	19	29.23%
No	46	70.77%
OCD diagnosis (76)		
Yes	19	25.00%
No	57	75.00%

Variable (n)	Mean	SD
Age, years (131)	15.65	1.40
Age of BDD onset, years (94)	12.30	2.39
Duration of BDD, years (94)	3.56	2.52
BDD-YBOCS-A total score (131)	34.73	5.46
CGAS (129)	41.00	8.76
AAI total score (114)	28.99	7.35
MFQ-C total score (75)	40.21	15.36
FAS-PR total score (131)	24.50	11.78
DASS total score (128)	29.99	26.76

*SD* Standard deviation, *ASD* autism spectrum disorder, *OCD* obsessive compulsive disorder, *BDD-YBOCS-A* body dysmorphic disorder-yale-brown obsessive–compulsive scale for adolescents, *CGAS* children’s global assessment scale, *AAI* appearance anxiety inventory, *MFQ-C* mood and feelings questionnaire child-version, *FAS-PR* family accommodation scale–parent report, *DASS* depression anxiety stress scale

### Frequency and Patterns of Maternal Accommodation

**Fig. 1:**
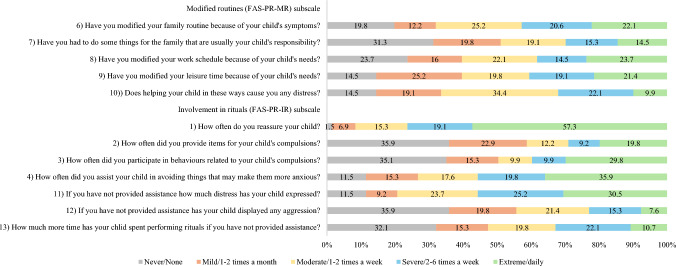
Percentage of mothers who selected each of the response options for each item of the Family Accommodation Scale–Parent Report (N = 131), with items of the FAS-PR separated by subscale

All mothers in the sample endorsed engaging in at least one form of accommodation on the FAS-PR, and the majority reported daily accommodation on at least one FAS-PR item (n = 97; 74%). As depicted in Fig. 1, providing reassurance, and assisting avoidance were the most endorsed accommodation behaviours.

### Demographic and Clinical Correlates of Maternal Accommodation

Maternal accommodation of BDD symptoms was not significantly correlated with child age and sex (p > 0.05). With regard to clinical correlates at assessment, the FAS-PR total score was significantly and positively correlated with clinician-rated BDD symptom severity (BDD-YBOCS-A; *r* = 0.18, *p* = 0.042; 95% CI [0.01, 0.34]) and maternal distress (DASS; *r* = 0.41, *p* < 0.001; 95% CI [0.26, 0.55]), and negatively correlated with global functioning (CGAS; *r* = − 0.38, *p* < 0.001; 95% CI [− 0.52, − 0.22]; Table 2). The FAS-PR total score was not significantly correlated with the self-report measure of BDD symptom severity and self-reported depressive symptoms. Interestingly, the correlation between the FAS-PR total score and self-reported BDD symptoms was small and of similar magnitude to the correlation between the FAS-PR total score and BDD clinician-rated BDD symptom severity, but did not reach statistical significance (AAI; *r* = 0.16, *p* = 0.09; 95% CI [− 0.02, 0.34]).

**Table 2:** Baseline maternal accommodation clinical correlates

	*r*		
Variable	FAS-PR total (95% CI)	FAS-PR-MR (95% CI)	FAS-PR-IR (95% CI)
BDD-YBOCS-A	0.179* (0.008, 0.340)	0.206* (0.036, 0.365)	0.135 (− 0.037, 0.300)
CGAS	− 0.375** (− 0.515, − 0.216)	− 0.435** (− 0.565, − 0.283)	− 0.277** (− 0.430, − 0.110)
AAI	0.162 (− 0.022, 0.336)	0.163 (− 0.022, 0.337)	0.151 (− 0.034, 0.326)
MFQ-C	0.180 (− 0.049, 0.391)	0.202 (− 0.026, 0.411)	0.138 (− 0.091, 0.354)
DASS	0.411** (0.255, 0.545)	0.372** (0.212, 0.512)	0.391** (0.233, 0.529)

*CI* Confidence interval, *FAS-PR* family accommodation scale–parent report, *FAS-PR-MR* family accommodation scale–parent report—modified routines subscale, *FAS-PR-IR* family accommodation scale–parent report—involvement in rituals subscale, *BDD-YBOCS-A* body dysmorphic disorder-yale-brown obsessive–compulsive scale for adolescents, *CGAS* children’s global assessment scale, *AAI* appearance anxiety inventory, *MFQ-C* mood and feelings questionnaire child-version, *DASS* depression anxiety stress scale

^*^correlation is significant at the 0.05 level (2-tailed)

^**^correlation is significant at the 0.01 level (2-tailed)

These correlation analyses were repeated with the two FAS-PR subscales (FAS-PR-MR and FAS-PR-IR) and a similar pattern of results was evident, such that associations with global functioning and maternal distress reached statistical significance for both subscales (Table 2). Clinician-rated BDD symptom severity was found to significantly positively correlate with the FAS-PR-MR subscale only (BDD-YBOCS-A; *r* = 0.21, *p* = 0.018; 95% CI [0.04, 0.37]).

A series of multiple linear regression analyses were conducted with the variables found to be significantly correlated with maternal accommodation in the bivariate analyses (Table 3), in order to determine their unique associations. Global functioning (CGAS) and maternal distress (DASS) showed unique and significant associations with maternal accommodation. Contrastingly, BDD symptom severity (BDD-YBOCS-A) did not show a unique and significant relationship with maternal accommodation.

**Table 3:** Summary of multiple linear regression models predicting maternal accommodation at assessment

Variable	FAS-PR total				FAS-PR-MR				FAS-PR-IR			
	β	SE	t	*p* value	β	SE	t	*p* value	β	SE	t	*p* value
CGAS	− 0.357	0.122	− 3.927	< 0.001	− 0.280	0.059	− 4.751	< 0.001	0.235	0.063	− 2.876	0.005
DASS	0.360	0.360	4.597	< 0.001	0.319	0.174	4.129	< 0.001	0.346	0.217	4.238	< 0.001
BDD-YBOCS-A	− 0.052	0.195	− 0.574	0.567	− 0.063	0.095	− 0.702	0.484	–	–	–	–
	R^2^ = 0.273, *p* < 0.001				R^2^ = 0.293, *p* < 0.001				R^2^ = 0.199, *p* < 0.001			

*SE* Standard error, *FAS-PR* family accommodation scale–parent report, *FAS-PR-MR* family accommodation scale–parent report—modified routines subscale, *FAS-PR-IR* family accommodation scale–parent report—involvement in rituals subscale, *CGAS* children’s global assessment scale, *DASS* depression anxiety stress scale, *BDD-YBOCS-A* body dysmorphic disorder-yale-brown obsessive–compulsive scale for adolescents, *SE* standard error

### Association Between Maternal Accommodation and Treatment Outcomes

A multiple linear regression was used to explore whether maternal accommodation at assessment significantly predicted post-treatment BDD symptom severity. The unadjusted model, which controlled for baseline BDD-YBOCS-A severity only, showed that the FAS-PR total score at assessment was not associated with post-treatment BDD-YBOCS-A (β = 0.055,  *p* = 0.588). The second model which adjusted for child age, sex, baseline BDD-YBOCS-A, CGAS and DASS, also showed that the FAS-PR total score at assessment was not associated with post-treatment BDD-YBOCS-A (β = 0.056, *p* = 0.650).

### Exploratory Post-Hoc Analyses

The somewhat paradoxical findings of high levels of maternal accommodation reported in this sample, yet a small correlation between maternal accommodation and BDD symptom severity at the univariate level only, and no evidence of an association between maternal accommodation and treatment outcomes, raised questions about what was driving accommodation. One possibility is that levels of maternal accommodation may have been somewhat accounted for by co-occurring conditions. In this sample there were high rates of OCD and Autism Spectrum Disorder (ASD), which have both previously been found to be associated with FA (e.g. [24, 45]). We therefore conducted post-hoc exploratory analyses which showed that young people with comorbid diagnoses of OCD and/or ASD had descriptively higher levels of maternal accommodation. Maternal accommodation was higher in young people with BDD and comorbid OCD (n = 13), ASD (n = 13), and OCD plus ASD (n = 6) compared to those with BDD and no OCD or ASD (n = 70, see Table S4). Next, the main correlation and regression analyses were repeated with the exclusion of BDD cases with a comorbid diagnosis of OCD and/or ASD. The pattern of results remained predominantly unchanged, with the exception of maternal accommodation (FAS-PR total and subscales) no longer being significantly correlated with maternal distress (DASS; see Table S5 and S6 of supplement).

Considering the finding that maternal accommodation was associated with BDD symptom severity at assessment but not post-treatment, we conducted a post-hoc analysis exploring change in maternal accommodation from assessment to post-treatment. In a subsample of participants with post-treatment FAS-PR data available (n = 47), the analysis suggested maternal accommodation reduced significantly from assessment (M = 24.70, SD = 12.17) to post-treatment (M = 17.91, SD = 12.10; t(46) = 3.026, *p* = 0.004). Given this significant reduction in maternal accommodation, a post-hoc multiple linear regression was used to explore whether changes in maternal accommodation from assessment to post-treatment (FAS-PR scores) significantly predicted post-treatment BDD symptom severity. The unadjusted model, which controlled for baseline BDD-YBOCS-A severity only, showed that greater changes in FAS-PR scores were significantly associated with reductions in in BDD symptom-severity (BDD-YBOCS-A) at post-treatment (β = − 0.382, *p* = 0.019). However, the second model, which adjusted for child age, sex, baseline BDD-YBOCS-A, CGAS and DASS, showed that changes in FAS-PR scores were not associated with post-treatment BDD-YBOCS-A (β = − 0.309, *p* = 0.141). Notably, there were no significant differences in sex, BDD severity (clinician and self-reported), global functioning, depressive symptoms, maternal accommodation, and maternal distress between participants who had FAS-PR post-treatment data available versus those who did not (all* p* > 0.05; see Table S7 of supplement). However, there was a significant difference in age, such that participants with post-treatment FAS-PR data available were younger (M = 15.26, SD = 1.57) than participants without post-treatment FAS-PR data available (M = 15.87, SD = 1.25; *p* = 0.024).

## Discussion

To our knowledge, this study provides the first quantitative investigation of maternal accommodation of BDD symptoms in young people. Three key findings emerged. First, maternal accommodation of BDD symptoms was common. Second, higher levels of maternal accommodation at assessment were weakly associated with greater clinician-rated BDD symptom severity, and moderately associated with greater maternal distress, and poorer child global functioning. Third, maternal accommodation of BDD symptoms at assessment did not predict outcomes following CBT for BDD.

In the current sample, all mothers reported engaging in at least one form of accommodation of their child’s BDD symptoms to some extent, with providing reassurance and facilitating avoidance being the most common. This finding is consistent with the qualitative accounts of parents of young people with BDD [31] and the high levels of FA consistently reported in studies with young people with a closely related condition, OCD (e.g. [21–23]). In a clinical cohort of young people accessing a specialist BDD clinic in Stockholm, Rautio et al. [12] found that 61.6% of young people reported seeking reassurance about their appearance concerns. Although reassurance seeking can take various forms in BDD, such as asking friends, searching online, and sharing content on social media, our findings suggest young people with BDD commonly seek and receive reassurance from their mothers.

As hypothesised, child age and sex were not associated with maternal accommodation. Regarding clinical correlates, we found that higher levels of maternal accommodation of BDD symptoms (FAS-PR total score [*r* = 0.18] and FAS-PR-MR subscale [*r* = 0.21]) were weakly associated with greater clinician-rated BDD symptom severity (BDD-YBOCS-A). Notably, although the relationship was significant, this finding should be interpreted with caution and warrants replication in future research, given the effect size was small and the relationship was only found to be significant at the univariate level. Research in OCD has shown a robust association between family accommodation (FA) and OCD symptom severity (e.g. [21, 22, 24]). Specifically, in their meta-analysis of 41 studies, Wu et al. [24] found a pooled effect size (*r*) of 0.42 for the relationship between FA and OCD symptom severity, although there was variation across studies, with effect sizes ranging from 0.10 to 0.82. The effect size observed in our study (*r* = 0.18) is therefore comparable to those reported by studies at the lower-end of the range of effect sizes in Wu et al.’s [24] meta-analysis. Post-hoc analyses revealed that young people with comorbid diagnoses of OCD and/or ASD had higher levels of maternal accommodation. Our results suggest that, unlike in OCD, the association between maternal accommodation and BDD may be primarily driven by factors other than BDD symptom severity.

In the previous qualitative study of FA and BDD, parents described a negative impact of FA on their own emotional wellbeing [31]. In line with this descriptive account, in the current study higher levels of maternal accommodation were significantly associated with greater maternal distress. In the field of paediatric OCD, a positive association has been found between maternal accommodation and maternal distress (e.g. [22, 26, 27]) and it has been suggested that mothers who experience their own psychological distress, particularly anxiety, may be more likely to seek to reduce their child’s distress, by accommodating symptoms [25, 27]. In this vein, it is plausible that mothers experiencing high levels of distress may be more prone to accommodating their child’s appearance-related behaviours. Alternatively, the opposite may be true, in that mothers may have experienced heightened distress as a consequence of the psychosocial burden of accommodating their child’s symptoms. In the current study, causal inferences cannot be made, but this warrants future research.

In the current study we found that higher levels of maternal accommodation of BDD symptoms were associated with worse child global functioning, consistent with findings in OCD (e.g. [22, 25, 26]). This finding could suggest that greater child global impairment could elicit increased accommodation of BDD symptoms from parents, for example, as they try to support their child to promote daily functioning. Alternatively, higher levels of maternal accommodation of BDD symptoms may limit the young person’s exposure to anxiety-provoking contexts and subsequently prevent opportunities to learn to tolerate and manage difficult emotions across settings, resulting in higher levels of impairment.

Lastly, in contrast to our hypothesis, our results show that maternal accommodation at assessment did not predict treatment outcomes. This finding was unexpected given the relatively consistent association demonstrated between higher levels of accommodation and worse treatment outcomes in paediatric OCD (e.g. [22, 28]). Cognitive-behavioural models of OCD formulate FA of symptoms as a compulsion which alleviates anxiety in the short-term but prevents opportunities for anxiety habituation and/or disconfirmation of feared outcomes, thereby fuelling OCD. Consequently, FA can obstruct the effectiveness of CBT, particularly E/RP components, unless successfully addressed in treatment [29, 30]. The current finding that maternal accommodation was associated with BDD symptom severity at assessment but not post-treatment, raises the possibility that maternal accommodation does not exert the same influence in the maintenance of BDD symptoms as found in OCD. For example, it is possible that young people with BDD engage in many compulsive behaviours (e.g. mirror checking) in private, without any parental involvement. Furthermore, we found that the most common form of maternal accommodation was provision of reassurance. It is possible that reassurance does not provide temporary anxiety relief, and therefore reassurance does not act as a reinforcing behavioural mechanism in the same way it does in OCD. Instead, reassurance seeking/provision may serve a different function in BDD. For example, in depression and health anxiety, reassurance seeking has been proposed to serve an interpersonal function regarding relationship security and care responses [46, 47]. Alternatively, in accordance with interpersonal models of depression, reassurance seeking in BDD could be conceptualised as negative feedback seeking, in which individuals are motivated by efforts to confirm their negative self-perceptions and consequently maintain a coherent sense of self [48]. Further understanding the functions and consequence of reassurance seeking in BDD is an important target for future research.

Additional possibilities are that exploring FA at the assessment may have increased the awareness mothers had of their accommodating behaviours, and in response to this, they may have changed these behaviours, or alternatively, maternal accommodation could have been successfully addressed in the intervention. In exploratory post-hoc analyses of a subsample with post-treatment FAS-PR data available (n = 47), maternal accommodation was found to significantly reduce from assessment to post-treatment, and greater changes in FAS-PR scores were significantly associated with a reduction in BDD symptom severity at post-treatment. Overall, however, it is important to consider that this subsample was small, and the direction of the association between change in maternal accommodation and BDD symptom severity remains unknown. Different hypotheses can be proposed, for example, as BDD symptoms improved, mothers may have consequently engaged in less accommodation, or as mothers reduced their accommodation, BDD symptoms may have subsequently improved, or alternatively, a third variable, such as maternal distress, could have related to reductions in both accommodation and BDD symptom severity. It therefore remains unclear why maternal accommodation reduced from assessment to post-treatment, and future research is warranted to explore mechanisms underpinning relationships between changes in maternal accommodation and BDD symptom severity. Whilst the treatment protocol used in this study does not explicitly address FA [10, 14], many of the clinicians, who are experts in treating BDD, may have targeted accommodating behaviours within the intervention. The involvement of mothers and targeting of accommodation was not measured in the study, therefore, an exploration of the extent to which clinicians are targeting accommodation in CBT for BDD is warranted in a larger sample to inform the development of existing interventions.

The current findings have clinical implications. First, the associations found between higher levels of maternal accommodation and greater BDD symptom severity and maternal distress, and worse global functioning suggest that during the assessment and treatment of adolescent BDD, clinicians should maintain an awareness of the ways in which mothers accommodate BDD and its influence on symptoms, and the broader impact on the lives of the young person and their mother. Specifically, BDD symptom severity was significantly associated with the ‘modified routines’ subscale of the FAS-PR but not the ‘involvement in rituals’ subscale. A reciprocal relationship between BDD severity and accommodation behaviours associated with modifying routines could be hypothesised, in which mothers change family routines and expectations of their child, and the child experiences a fear of being negatively judged or rejected by others based on their appearance, and as a result they withdraw from social interactions and reduce their participation in family life. In contrast, a young person may carry out BDD-related routines in private (e.g. mirror checking, grooming routines) due to shame and embarrassment, meaning that mothers do not become actively involved in such behaviours. Consequently, it could be helpful for clinicians to specifically explore accommodation behaviours which are associated with family functioning, and the child and parental beliefs underpinning these behaviours. Second, at present, CBT for BDD treatment protocols do not explicitly address FA [10, 14] and the extent of parental involvement is dependent upon the individual case formulation and developmental needs of the young person. The literature on FA in OCD highlights it is a significant factor that is often targeted in interventions by including parents in CBT (e.g. [22, 29, 30]). In contrast, there is no clarity of if, or how, parents should or could be involved in CBT for BDD for adolescents. The results of the current study imply that to improve existing treatments, assumptions should not be made that approaches necessary and beneficial for working with young people with OCD, will also be required and helpful when supporting young people with BDD. Although the two disorders share overlapping features in their presentation, diagnosis and treatment, OCD and BDD are distinct conditions that require separate research, understanding, and support in clinical practice.

Limitations of the study must be considered whilst interpreting the current findings. First, the generalisability of findings may be hindered by the characteristics of the sample, for example, most of the young people identified as White British and all participants were from one national and specialist clinic in the United Kingdom where young people often present with severe and long-standing BDD symptoms. Additionally, although no significant differences were found between participants with and without post-treatment data (clinician-administered and mother-reported) for most of the clinical and demographic characteristics measured, females were found to be significantly more likely than males to have clinician-administered post-treatment data available, and participants with mother-reported post-treatment data were significantly younger than participants without mother-reported post-treatment data. Taken together, these differences could be considered to limit the generalisability of findings from the subsamples of those with clinician-administered and mother-reported post-treatment data.

Second, comorbid diagnoses were not systematically and formally assessed and therefore the role of comorbidities in maternal accommodation and its associations remains poorly understood and requires further exploration. Third, this study utilised mother-reported data only, and therefore the patterns and correlates of father-reported accommodation in adolescent BDD remain unknown, and warrant exploration. Fourth, maternal accommodation was assessed using a self-report measure which may have been subject to bias [20]. Future research could seek to include multi-informant assessment of FA, such as structured observations, clinician-rated instruments (e.g. FAS, [41], Paediatric Accommodation Scale, [49]) and child-rated measures (e.g. see FA Scale for Anxiety—Child report, [50]). Furthermore, given variability exists in levels of parent-reported FA at assessment, future research exploring associations between FA and treatment outcomes using larger samples could undertake subgroup analyses of participants with high and low levels of FA. Fifth, maternal involvement in treatment and the targeting of FA during treatment were not systematically measured. Therefore, the impact of the extent to which mothers were involved in treatment and/or accommodation behaviours were explicitly addressed on patterns and correlates of maternal accommodation, and its association with treatment outcomes, is unknown.

## Summary

The current study provides a quantitative exploration of maternal accommodation of adolescent BDD. The results show that maternal accommodation of BDD symptoms was ubiquitous in this sample. Maternal accommodation was only weakly associated with BDD symptom severity, suggesting that other factors, such as psychiatric comorbidity, maternal distress and impairments in global functioning may be more important contributors. Maternal accommodation was not significantly associated with treatment outcomes. Consequently, it appears the relationship between maternal accommodation and BDD symptoms may differ to that evidenced in paediatric OCD. Longitudinal research exploring both maternal and paternal accommodation, utilising measures completed by multiple informants, and assessing variables of interest at multiple time-points throughout treatment, is needed to further our understanding of the role of FA in adolescent BDD and how to approach FA in CBT for BDD with adolescents.

## Supplementary Information

Below is the link to the electronic supplementary material.

Supplementary file1 (DOCX 53 KB)

## Data Availability

Data is provided within the manuscript or supplementary information files.

## References

[1] American Psychiatric Association. (2013). Diagnostic and statistical manual of mental disorders: DSM-5.

[2] World Health Organization. (2018). International statistical classification of diseases and related health problems (11th Revision). 10.2471/BLT.25.294650 PMC13531092 42683092

[3] Bjornsson AS, Didie ER, Grant JE, Menard W, Stalker E, Phillips KA (2013) Age at onset and clinical correlates in body dysmorphic disorder. Compr Psychiatry 54(7):893–903. 10.1016/j.comppsych.2013.03.019 23643073 10.1016/j.comppsych.2013.03.019 PMC3779493

[4] Krebs G, Clark B, Ford T, Stringaris A (2023) Epidemiology of body dysmorphic disorder and appearance preoccupation in youth: prevalence, comorbidity and psychosocial impairment. J Am Acad Child Adolesc Psychiatry. 10.31234/osf.io/zmd2h 10.1016/j.jaac.2024.01.017 38508411

[5] Möllmann A, Dietel FA, Hunger A, Buhlmann U (2017) Prevalence of body dysmorphic disorder and associated features in German adolescents: a self-report survey. Psychiatry Res 254:263–267. 10.1016/j.psychres.2017.04.063 28482195 10.1016/j.psychres.2017.04.063

[6] Veale D, Gledhill LJ, Christodoulou P, Hodsoll J (2016) Body dysmorphic disorder in different settings: a systematic review and estimated weighted prevalence. Body Image 18:168–186. 10.1016/j.bodyim.2016.07.003 27498379 10.1016/j.bodyim.2016.07.003

[7] Angelakis I, Gooding PA, Panagioti M (2016) Suicidality in body dysmorphic disorder (BDD): A systematic review with meta-analysis. Clin Psychol Rev 49:55–66. 10.1016/j.cpr.2016.08.002 27607741 10.1016/j.cpr.2016.08.002

[8] Didie ER, Menard W, Stern AP, Phillips KA (2008) Occupational functioning and impairment in adults with body dysmorphic disorder. Compr Psychiatry 49(6):561–569. 10.1016/j.comppsych.2008.04.003 18970904 10.1016/j.comppsych.2008.04.003

[9] Krebs G, de la Cruz LF, Rijsdijk FV, Rautio D, Enander J, Rück C, Mataix-Cols D (2020) The association between body dysmorphic symptoms and suicidality among adolescents and young adults: a genetically informative study. Psychol Med 52(7):1268–1276. 10.1017/S0033291720002998 32940195 10.1017/S0033291720002998 PMC9157307

[10] Mataix-Cols D, de la Cruz LF, Isomura K, Anson M, Turner C, Monzani B, Krebs G (2015) A pilot randomized controlled trial of cognitive-behavioral therapy for adolescents with body dysmorphic disorder. J Am Acad Child Adolesc Psychiatry 54(11):895–904. 10.1016/j.jaac.2015.08.011 26506580 10.1016/j.jaac.2015.08.011

[11] Phillips KA, Menard W, Fay C, Pagano ME (2005) Psychosocial functioning and quality of life in body dysmorphic disorder. Compr Psychiatry 46(4):254–260. 10.1016/j.comppsych.2004.10.004 16175755 10.1016/j.comppsych.2004.10.004 PMC1351256

[12] Rautio D, Jassi A, Krebs G, Andrén P, Monzani B, Gumpert M, Mataix-Cols D (2020) Clinical characteristics of 172 children and adolescents with body dysmorphic disorder. Eur Child Adolesc Psychiatry. 10.1007/s00787-020-01677-3 33165651 10.1007/s00787-020-01677-3 PMC8817062

[13] National Institute for Health and Care Excellence. (2005). Obsessive-compulsive disorder and body dysmorphic disorder: treatment [NICE Guideline CG31]. https://www.nice.org.uk/guidance/cg31 39480980

[14] Rautio D, Gumpert M, Jassi A, Krebs G, Flygare O, Andrén P, Mataix-Cols D (2022) Effectiveness of multimodal treatment for young people with body dysmorphic disorder in two specialist clinics. Behav Ther 53(5):1037–1049. 10.1016/j.beth.2022.04.010 35987534 10.1016/j.beth.2022.04.010

[15] Flygare O, Enander J, Andersson E, Ljótsson B, Ivanov VZ, Mataix-Cols D, Rück C (2020) Predictors of remission from body dysmorphic disorder after internet-delivered cognitive behavior therapy: a machine learning approach. BMC Psychiatry 20:1–9. 10.1186/s12888-020-02655-4 32429939 10.1186/s12888-020-02655-4 PMC7238519

[16] Greenberg JL, Phillips KA, Steketee G, Hoeppner SS, Wilhelm S (2019) Predictors of response to cognitive-behavioral therapy for body dysmorphic disorder. Behav Ther 50(4):839–849. 10.1016/j.beth.2018.12.008 31208692 10.1016/j.beth.2018.12.008 PMC6582981

[17] Harrison A, de la Cruz LF, Enander J, Radua J, Mataix-Cols D (2016) Cognitive-behavioral therapy for body dysmorphic disorder: A systematic review and meta-analysis of randomized controlled trials. Clin Psychol Rev 48:43–51. 10.1016/j.cpr.2016.05.007 27393916 10.1016/j.cpr.2016.05.007

[18] Hogg E, Adamopoulos P, Krebs G (2023) Predictors and moderators of treatment response in cognitive behavioural therapy for body dysmorphic disorder: a systematic review. J Obsessive-Compul Relat Disord. 10.1016/j.jocrd.2023.100822

[19] Phillips KA, Greenberg JL, Hoeppner SS, Weingarden H, O’Keefe S, Keshaviah A, Wilhelm S (2021) Predictors and moderators of symptom change during cognitive-behavioral therapy or supportive psychotherapy for body dysmorphic disorder. J Affect Disord 287:34–40. 10.1016/j.jad.2021.03.011 33773357 10.1016/j.jad.2021.03.011 PMC8276884

[20] Kagan ER, Frank HE, Kendall PC (2017) Accommodation in youth with OCD and anxiety. Clin Psychol Sci Pract 24(1):78. 10.1111/cpsp.12186

[21] Jacoby RJ, Smilansky H, Shin J, Wu MS, Small BJ, Wilhelm S, Geller DA (2021) Longitudinal trajectory and predictors of change in family accommodation during exposure therapy for pediatric OCD. J Anxiety Disord 83:102463. 10.1016/j.janxdis.2021.102463 34428688 10.1016/j.janxdis.2021.102463 PMC8925412

[22] Monzani B, Vidal-Ribas P, Turner C, Krebs G, Stokes C, Heyman I, Stringaris A (2020) The role of paternal accommodation of paediatric OCD symptoms: patterns and implications for treatment outcomes. J Abnorm Child Psychol 48:1313–1323. 10.1007/s10802-020-00678-9 32683586 10.1007/s10802-020-00678-9 PMC7445192

[23] Wu MS, Geller DA, Schneider SC, Small BJ, Murphy TK, Wilhelm S, Storch EA (2019) Comorbid psychopathology and the clinical profile of family accommodation in pediatric OCD. Child Psychiatry Hum Dev 50:717–726. 10.1007/s10578-019-00876-7 30790098 10.1007/s10578-019-00876-7 PMC6703960

[24] Wu MS, McGuire JF, Martino C, Phares V, Selles RR, Storch EA (2016) A meta-analysis of family accommodation and OCD symptom severity. Clin Psychol Rev 45:34–44. 10.1016/j.cpr.2016.03.003 27019367 10.1016/j.cpr.2016.03.003

[25] Caporino NE, Morgan J, Beckstead J, Phares V, Murphy TK, Storch EA (2012) A structural equation analysis of family accommodation in pediatric obsessive-compulsive disorder. J Abnorm Child Psychol 40:133–143. 10.1007/s10802-011-9549-8 21842196 10.1007/s10802-011-9549-8

[26] Pontillo M, Demaria F, Tata MC, Averna R, Gargiullo P, Pucciarini ML, Vicari S (2020) Clinical significance of family accommodation and parental psychological distress in a sample of children and adolescents with obsessive-compulsive disorder aged 8–17 years old. Ital J Pediatr 46:1–10. 10.1186/s13052-020-00932-2 33168039 10.1186/s13052-020-00932-2 PMC7654062

[27] Flessner CA, Freeman JB, Sapyta J, Garcia A, Franklin ME, March JS, Foa E (2011) Predictors of parental accommodation in pediatric obsessive-compulsive disorder: Findings from the pediatric obsessive-compulsive disorder treatment study (POTS) trial. J Am Acad Child Adolesc Psychiatry 50(7):716–725. 10.1016/j.jaac.2011.03.019 21703499 10.1016/j.jaac.2011.03.019 PMC3128390

[28] Turner C, O’Gorman B, Nair A, O’Kearney R (2018) Moderators and predictors of response to cognitive behaviour therapy for pediatric obsessive-compulsive disorder: a systematic review. Psychiatry Res 261:50–60. 10.1016/j.psychres.2017.12.034 29287236 10.1016/j.psychres.2017.12.034

[29] Peris TS, Bergman RL, Langley A, Chang S, McCracken JT, Piacentini J (2008) Correlates of accommodation of pediatric obsessive-compulsive disorder: parent, child, and family characteristics. J Am Acad Child Adolesc Psychiatry 47(10):1173–1181. 10.1097/CHI.0b013e3181825a91 18724255 10.1097/CHI.0b013e3181825a91 PMC3378323

[30] Storch EA, Geffken GR, Merlo LJ, Jacob ML, Murphy TK, Goodman WK, Grabill K (2007) Family accommodation in pediatric obsessive–compulsive disorder. J Clin Child Adolesc Psychol 36(2):207–216. 10.1080/15374410701277929 17484693 10.1080/15374410701277929

[31] Jassi AD, Baloch A, Thomas-Smith K, Lewis A (2020) Family accommodation in pediatric body dysmorphic disorder: a qualitative study. Bull Menninger Clin 84(4):319–336. 10.1521/bumc.2020.84.4.319 33779234 10.1521/bumc.2020.84.4.319

[32] Phillips KA, Hollander E, Rasmussen SA, Aronowitz BR (1997) A severity rating scale for body dysmorphic disorder: development, reliability, and validity of a modified version of the yale-brown obsessive compulsive scale. Psychopharmacol Bull 33(1):17 9133747

[33] Shaffer D, Gould MS, Brasic J, Ambrosini P, Fisher P, Bird H, Aluwahlia S (1983) A children’s global assessment scale (CGAS). Arch Gen Psychiatry 40(11):1228–1231. 10.1001/archpsyc.1983.01790100074010 6639293 10.1001/archpsyc.1983.01790100074010

[34] Bird HR, Canino G, Rubio-Stipec M, Ribera JC (1987) Further measures of the psychometric properties of the children’s global assessment scale. Arch Gen Psychiatry 44(9):821–824. 10.1001/archpsyc.1987.01800210069011 3632256 10.1001/archpsyc.1987.01800210069011

[35] Veale D, Eshkevari E, Kanakam N, Ellison N, Costa A, Werner T (2014) The appearance anxiety inventory: validation of a process measure in the treatment of body dysmorphic disorder. Behav Cogn Psychother 42(5):605–616. 10.1017/S1352465813000556 23823485 10.1017/S1352465813000556

[36] Gumpert M, Rautio D, Monzani B, Jassi A, Krebs G, Fernández de la Cruz L, Jansson-Fröjmark M (2024) Psychometric evaluation of the appearance anxiety inventory in adolescents with body dysmorphic disorder. Cogn Behav Ther. 10.1080/16506073.2023.2299837 38174353 10.1080/16506073.2023.2299837

[37] Mastro S, Zimmer-Gembeck MJ, Webb HJ, Farrell L, Waters A (2016) Young adolescents’ appearance anxiety and body dysmorphic symptoms: social problems, self-perceptions and comorbidities. J Obsessive-Compul Relat Disord 8:50–55. 10.1016/j.jocrd.2015.12.001

[38] Burleson Daviss W, Birmaher B, Melhem NA, Axelson DA, Michaels SM, Brent DA (2006) Criterion validity of the mood and feelings questionnaire for depressive episodes in clinic and non-clinic subjects. J Child Psychol Psychiatry 47(9):927–934. 10.1111/j.1469-7610.2006.01646.x 16930387 10.1111/j.1469-7610.2006.01646.x

[39] Sund AM, Larsson B, Wichstrøm L (2001) Depressive symptoms among young Norwegian adolescents as measured by the Mood and Feelings Questionnaire (MFQ). Eur Child Adolesc Psychiatry 10:222–229. 10.1007/s007870170011 11794547 10.1007/s007870170011

[40] Flessner CA, Sapyta J, Garcia A, Freeman JB, Franklin ME, Foa E, March J (2011) Examining the psychometric properties of the family accommodation scale-parent-report (FAS-PR). J Psychopathol Behav Assess 33:38–46. 10.1007/s10862-010-9196-3 10.1007/s10862-010-9196-3 PMC3131184 21743772

[41] Calvocoressi L, Lewis B, Harris M, Trufan SJ, Goodman WK, McDougle CJ, Price LH (1995) Family accommodation in obsessive-compulsive disorder. Am J Psychiatry. 10.1176/ajp.152.3.441 7864273 10.1176/ajp.152.3.441

[42] Lovibond PF, Lovibond SH (1995) The structure of negative emotional states: comparison of the Depression Anxiety Stress Scales (DASS) with the beck depression and anxiety inventories. Behav Res Ther 33(3):335–343. 10.1016/0005-7967(94)00075-U 7726811 10.1016/0005-7967(94)00075-u

[43] Faul F, Erdfelder E, Buchner A, Lang AG (2009) Statistical power analyses using G* Power 3.1: tests for correlation and regression analyses. Behav Res Method 41(4):1149–1160. 10.3758/BRM.41.4.1149 10.3758/BRM.41.4.1149 19897823

[44] Siddiqui OI (2015) Methods for computing missing item response in psychometric scale construction. Am J Biostat 5(1):1. 10.3844/amjbsp.2015.1.6

[45] Feldman I, Koller J, Lebowitz ER, Shulman C, Ben Itzchak E, Zachor DA (2019) Family accommodation in autism spectrum disorder. J Autism Dev Disord 49:3602–3610. 10.1007/s10803-019-04078-x 31134428 10.1007/s10803-019-04078-x

[46] Birnie KA, Sherry SB, Doucette S, Sherry DL, Hadjistavropoulos HD, Stewart SH (2013) The interpersonal model of health anxiety: testing predicted paths and model specificity. Personality Individ Differ 54(7):856–861. 10.1016/j.paid.2012.12.028

[47] Coyne JC (1976) Toward an interactional description of depression. Psychiatry 39(1):28–40. 10.1080/00332747.1976.11023874 1257353 10.1080/00332747.1976.11023874

[48] Joiner TE Jr, Alfano MS, Metalsky GI (1993) Caught in the crossfire: depression, self-consistency, self-enhancement, and the response of others. J Soc Clin Psychol 12(2):113–134. 10.1521/jscp.1993.12.2.113

[49] Benito KG, Caporino NE, Frank HE, Ramanujam K, Garcia A, Freeman J, Storch EA (2015) Development of the pediatric accommodation scale: reliability and validity of clinician-and parent-report measures. J Anxiety Disord 29:14–24. 10.1016/j.janxdis.2014.10.004 25481401 10.1016/j.janxdis.2014.10.004

[50] Lebowitz ER, Scharfstein LA, Jones J (2014) Comparing family accommodation in pediatric obsessive-compulsive disorder, anxiety disorders, and nonanxious children. Depress Anxiety 31(12):1018–1025. 10.1002/da.22251 24677578 10.1002/da.22251

