# Supplementary material for: Maternal Accommodation of Adolescent Body Dysmorphic Disorder: Clinical Correlates and Association with Treatment Outcomes
Source: Child Psychiatry Hum Dev. 2024 Sep 3;57(3):881–92. doi: 10.1007/s10578-024-01754-7 (PMC13201311; doi:10.1007/s10578-024-01754-7)
Supplement: Supplementary file 1 — Supplementary file1 (DOCX 53 KB) [file 10578_2024_1754_MOESM1_ESM.docx]

# Supplementary material

**Figure S1**

*Exploratory Factor Analysis of the Family Accommodation Scale – Parent Report: Scree plot*

**Table S1**

*t-tests and chi-squared test comparing demographic and clinical variables for participants with and without post-treatment BDD-YBOCS-A data available*

|  | **Post-treatment BDD-YBOCS-A data available** | | | **Post-treatment BDD-YBOCS-A data unavailable** | | |  |  |
| --- | --- | --- | --- | --- | --- | --- | --- | --- |
| **Variable** | **n** | **Mean** | **SD** | **n** | **Mean** | **SD** | **t** | ***p*** |
| **Age, years** | 76 | 15.55 | 1.31 | 55 | 15.78 | 1.47 | .926 | .37 |
| **BDD-YBOCS-A Total score** | 76 | 35.43 | 5.30 | 55 | 33.76 | 5.56 | -1.743 | .084 |
| **CGAS** | 75 | 40.76 | 8.74 | 54 | 41.33 | 8.87 | .365 | .715 |
| **AAI Total score** | 67 | 29.13 | 9.94 | 47 | 28.76 | 7.98 | -2.62 | .794 |
| **MFQ-C Total score** | 39 | 40.23 | 16.44 | 36 | 40.19 | 14.32 | -.010 | .992 |
| **FAS-PR Total score** | 76 | 23.64 | 12.03 | 55 | 25.69 | 11.42 | .981 | .328 |
| **DASS Total score** | 74 | 28.16 | 26.90 | 54 | 32.50 | 26.59 | .905 | .367 |
|  | **Female (n)** | **Male (n)** |  | **Female (n)** | **Male (n)** |  | **Χ^2^** | ***p*** |
| **Sex** | 67 | 9 |  | 41 | 14 |  | 4.085 | .043 |

Note: SD, standard deviation; BDD-YBOCS-A, Body Dysmorphic Disorder-Yale-Brown Obsessive-Compulsive Scale for Adolescents; CGAS, Children’s Global Assessment Scale; AAI, Appearance Anxiety Inventory; MFQ-C, Mood and Feelings Questionnaire Child-Version; FAS-PR, Family Accommodation Scale - Parent Report; DASS, Depression Anxiety Stress Scale.

**Table S2**

*Exploratory Factor Analysis of the Family Accommodation Scale – Parent Report: retained items and two-factor solution factor loadings*

|  | Factor loadings | |
| --- | --- | --- |
|  | I | II |
| **Factor 1 – Modified routines (FAS-PR-MR)** |  |  |
| 8) Have you modified your work schedule because of your child’s needs? | 1.012 | -.219 |
| 9) Have you modified your leisure time because of your child’s needs? | .948 | -.084 |
| 6) Have you modified your family routine because of your child’s symptoms? | .724 | -174 |
| 10) Does helping your child in these ways cause you any distress? | .529 | .228 |
| 7) Have you had to do some things for the family that are usually your child’s responsibility? | .409 | .308 |
| **Factor 2 – Involvement in rituals (FAS-PR-IR)** |  |  |
| 2) How often did you provide items for your child’s compulsions? | -.040 | .694 |
| 11) If you have not provided assistance how much distress has your child expressed? | .212 | .672 |
| 3) How often did you participate in behaviours related to your child’s compulsions? | .051 | .610 |
| 13) How much more time has your child spent performing rituals if you have not provided assistance? | .149 | .596 |
| 4) How often did you assist your child in avoiding things that may make them more anxious? | .187 | .545 |
| 12) If you have not provided assistance has your child displayed any aggression? | .051 | .526 |
| 1) How often do you reassure your child? | -.157 | .490 |

**Table S3**

*t-tests and chi-squared test comparing demographic and clinical variables for participants taking SSRI medication during treatment and those not taking SSRI medication during treatment*

|  | **Taking SSRI medication** | | | **Not taking SSRI medication** | | |  |  |
| --- | --- | --- | --- | --- | --- | --- | --- | --- |
| **Variable** | **n** | **Mean** | **SD** | **n** | **Mean** | **SD** | **t** | ***p*** |
| **Age, years** | 40 | 15.75 | 1.21 | 29 | 15.34 | 1.61 | -1.192 | .260 |
| **BDD-YBOCS-A Total score** | 40 | 34.33 | 5.10 | 29 | 36.66 | 5.07 | 1.879 | .065 |
| **CGAS** | 39 | 40.56 | 7.94 | 29 | 41.38 | 10.29 | .355 | .724 |
| **AAI Total score** | 34 | 29.56 | 6.35 | 26 | 28.66 | 7.80 | -.496 | .622 |
| **MFQ-C Total score** | 27 | 41.74 | 16.03 | 6 | 36.50 | 18.84 | -.703 | .487 |
| **FAS-PR Total score** | 40 | 24.78 | 11.48 | 29 | 21.28 | 12.04 | -1.213 | .229 |
| **DASS Total score** | 39 | 32.21 | 30.63 | 28 | 25.21 | 24.88 | -.994 | .324 |
|  | **Female (n)** | **Male (n)** |  | **Female (n)** | **Male (n)** |  | **Χ^2^** | ***p*** |
| **Sex** | 34 | 6 |  | 25 | 4 |  | .020 | .888 |

Note: SD, standard deviation; BDD-YBOCS-A, Body Dysmorphic Disorder-Yale-Brown Obsessive-Compulsive Scale for Adolescents; CGAS, Children’s Global Assessment Scale; AAI, Appearance Anxiety Inventory; MFQ-C, Mood and Feelings Questionnaire Child-Version; FAS-PR, Family Accommodation Scale - Parent Report; DASS, Depression Anxiety Stress Scale.

**Table S4**

*Sample size and descriptives for levels of maternal accommodation and associated variables of interest for subgroups of the sample based upon comorbid diagnoses (where data was available)*

|  | | **FAS-PR** | | **BDD-YBOCS-A** | | **CGAS** | | **DASS** | |
| --- | --- | --- | --- | --- | --- | --- | --- | --- | --- |
|  | **n** | **Mean** | **SD** | **Mean** | **SD** | **Mean** | **SD** | **Mean** | **SD** |
| **All sample** | 131 | 24.25 | 24.25 | 34.85 | 5.60 | 41.00 | 8.76 | 29.99 | 26.76 |
| **BDD only** | 70 | 22.96 | 11.18 | 34.04 | 5.14 | 42.24 | 8.97 | 30.26 | 26.92 |
| **BDD and ASD** | 13 | 29.62 | 11.98 | 35.23 | 6.95 | 39.85 | 9.65 | 34.25 | 23.49 |
| **BDD and OCD** | 13 | 28.40 | 11.21 | 32.77 | 4.94 | 40.08 | 8.36 | 32.46 | 33.91 |
| **BDD and ASD and OCD** | 6 | 39.67 | 39.67 | 33.83 | 5.98 | 37.00 | 4.82 | 33.00 | 20.07 |

Note: ASD, Autism Spectrum Disorder; OCD, Obsessive Compulsive Disorder; FAS-PR, Family Accommodation Scale - Parent Report; BDD-YBOCS-A, Body Dysmorphic Disorder-Yale-Brown Obsessive-Compulsive Scale for Adolescents; CGAS, Children’s Global Assessment Scale; DASS, Depression Anxiety Stress Scale

**Table S5**

*Baseline maternal accommodation clinical correlates with the exclusion of cases with a diagnosis of OCD and/or ASD (n=70)*

|  | ***r*** | | |
| --- | --- | --- | --- |
| **Variable** | **FAS-PR Total**  **(95% CI)** | **FAS-PR-MR**  **(95% CI)** | **FAS-PR-IR**  **(95% CI)** |
| **BDD-YBOCS-A** | .244*  (.009, .453) | .256*  (.022, .463) | .199  (-.037, .415) |
| **CGAS** | -.512**  (-.667, -.315) | -.573**  (-.712, -.391) | -.388*  (-.571, -.168) |
| **AAI** | .004  (-.257, .264) | .052  (-.211, .308) | -.040  (-.298, .223) |
| **MFQ-C** | .127  (-.154, .389) | .126  (-.155, .388) | .112  (-.169, .376) |
| **DASS** | .196  (-.043, .413) | .174  (-.065, .384) | .186  (-.053, .405) |

Note: *correlation is significant at the 0.05 level (2-tailed); **correlation is significant at the 0.01 level (2-tailed); CI, Confidence Interval; FAS-PR, Family Accommodation Scale - Parent Report; FAS-PR-MR, Family Accommodation Scale - Parent Report – Modified Routines subscale; FAS-PR-IR, Family Accommodation Scale - Parent Report – Involvement in Rituals subscale; BDD-YBOCS-A, Body Dysmorphic Disorder-Yale-Brown Obsessive-Compulsive Scale for Adolescents; CGAS, Children’s Global Assessment Scale; AAI, Appearance Anxiety Inventory; MFQ-C, Mood and Feelings Questionnaire Child-Version; DASS, Depression Anxiety Stress Scale

**Table S6**

*Multiple linear regression model of baseline family accommodation predicting treatment outcomes with the exclusion of cases with a diagnosis of OCD and/or ASD (n=40)*

| **Assessment variable** | **Post-treatment BDD-YBOCS-A** | | |
| --- | --- | --- | --- |
|  | **β** | **SE** | **t** |
| **FAS-PR Total** | .171 | .154 | 1.108 |
|  | R^2^=.287, *p*=.066 | | |

Note: SE, Standard Error; FAS-PR, Family Accommodation Scale - Parent Report; FAS-PR-MR, Family Accommodation Scale - Parent Report – Modified Routines subscale; FAS-PR-IR, Family Accommodation Scale - Parent Report – Involvement in Rituals subscale; CGAS, Children’s Global Assessment Scale; DASS, Depression Anxiety Stress Scale; BDD-YBOCS-A, Body Dysmorphic Disorder-Yale-Brown Obsessive-Compulsive Scale for Adolescents; SE, standard error. The model controlled for baseline BDD severity (BDD-YBOCS-A), child age, child sex and baseline CGAS.

**Table S7**

*t-tests and chi-squared test comparing demographic and clinical variables for participants with and without post-treatment FAS-PR data available*

|  | **Post-treatment FAS-PR data available** | | | **Post-treatment FAS-PR data unavailable** | | |  |  |
| --- | --- | --- | --- | --- | --- | --- | --- | --- |
| **Variable** | **n** | **Mean** | **SD** | **n** | **Mean** | **SD** | **t** | ***p*** |
| **Age, years** | 47 | 15.26 | 1.57 | 84 | 15.87 | 1.25 | 2.306 | .024 |
| **BDD-YBOCS-A Total score** | 47 | 35.60 | 6.25 | 84 | 34.25 | 4.93 | -1.358 | .177 |
| **CGAS** | 45 | 39.69 | 8.38 | 84 | 39.69 | 8.29 | 1.246 | .215 |
| **AAI Total score** | 41 | 28.59 | 8.43 | 73 | 28.59 | 8.43 | .431 | .668 |
| **MFQ-C Total score** | 15 | 37.27 | 17.84 | 60 | 37.27 | 17.84 | .829 | .410 |
| **FAS-PR Total score** | 47 | 24.70 | 12.17 | 84 | 24.39 | 11.63 | -.144 | .886 |
| **DASS Total score** | 64 | 27.48 | 25.29 | 82 | 31.40 | 27.56 | .795 | .428 |
|  | **Female (n)** | **Male (n)** |  | **Female (n)** | **Male (n)** |  | **Χ^2^** | ***p*** |
| **Sex** | 42 | 66 |  | 5 | 18 |  | 2.424 | .119 |

Note: SD, standard deviation; BDD-YBOCS-A, Body Dysmorphic Disorder-Yale-Brown Obsessive-Compulsive Scale for Adolescents; CGAS, Children’s Global Assessment Scale; AAI, Appearance Anxiety Inventory; MFQ-C, Mood and Feelings Questionnaire Child-Version; FAS-PR, Family Accommodation Scale - Parent Report; DASS, Depression Anxiety Stress Scale.
